# TFAP2A Induced ITPKA Serves as an Oncogene and Interacts with DBN1 in Lung Adenocarcinoma

**DOI:** 10.7150/ijbs.40435

**Published:** 2020-01-01

**Authors:** Zhou Guoren, Fan Zhaohui, Zhu Wei, Wang Mei, Wu Yuan, Shi Lin, Xu Xiaoyue, Zhang Xiaomei, Shen Bo

**Affiliations:** 1 Jiangsu Cancer Hospital, Jiangsu Institute Of Cancer Research, Nanjing Medical University Affiliated Cancer Hospital; 42 Baiziting, Nanjing, Jiangsu, 210009, China (Corresponding Address); 2 School Of Medicine, Jiangsu University, Zhenjiang, Jiangsu, China

**Keywords:** TFAP2A, ITPKA, LUAD, EMT, Drebrin 1

## Abstract

The inositol polyphosphate kinase (IPK) family member ITPKA (inositol 1,4,5-trisphosphate 3-kinase) regulates the levels of many inositol polyphosphates which are important in cellular signaling. Several recent studies reported the aberrant expression of ITPKA in malignancy disease and usually made cancer more aggressive. However, the contribution of the inositol polyphosphate kinase ITPKA to lung cancer development remains unclear.

Here we report that ITPKA is overexpressed in lung adenocarcinoma (LUAD) and positively correlated with advanced clinical parameters. ITPKA contributes to the malignant phenotypes *in-vitro*. Mechanistically, ITPKA executed its action through the inducting of epithelial-mesenchymal transition (EMT) and interacting with Drebrin 1 (which is related to cancer metastasis). Moreover, the hyper-expression of ITPKA in LUAD is transcriptionally activated by the transcription factor TFAP2A. In survival analysis by using tissue microarray (TMA), we indicate that ITPKA is hyper-expressed in LUAD tissues compared to adjacent normal tissues, and increased expression of ITPKA is associated with poor prognosis.

Collectively, this study indicates that TFAP2A induced ITPKA hyperexpression promotes LUAD via interacting with Drebrin 1 and activating epithelial-mesenchymal transition (EMT). ITPKA might represent a potent candidate for the treatment and prognostic prediction of LUAD.

## Introduction

The Inositol-trisphosphate 3-kinase (InsP3-kinase) proteins are enzymes belongs to the family of transferases which facilitates phospho-group transfer from adenosine triphosphate to 1D-myo-inositol 1,4,5-trisphosphate 1. The InsP3-kinase family contains three isoforms, ITPKA, ITPKB, and ITPKC; these three isoforms contain the same conserved C-terminal catalytic domain but differ in mechanisms of regulation as well as tissue expression.

Neuron-specific F-actin bundling protein InsP3-kinase-A (ITPKA) contributes to the formation of cellular protrusions which is a prerequisite for cells to migrate, the actin-modulating activity of ITPKA increases the migratory and the metastatic potential of tumor cells 2, 3. Windhorst et al. reported that high expression of ITPKA increases the motility of tumor cells and increases the metastatic potential of malignant cells in cancer patients 4, 5. Moreover, ITPKA was limited in tissue distribution and identified as a potential oncogene, normally ITPKA mainly expressed in the brain, but recently the aberrant hyper-expression of ITPKA were found in many malignant diseases and associated with metastasis 4, 6, 7, thus ITPKA could be an excellent target for selective therapy for malignant diseases.

To explore the role of ITPKA hyper-expression in tumors, Wang et al. reported that the ITPKA gene body methylation regulates gene expression via modulation of the binding of SP1 transcription factor to the ITPKA promoter 6. Chang et al. reported that the repressor-element-1-silencing transcription factor (REST)/neuron-restrictive silencer factor (NRSF), which suppresses the expression of neuronal genes in nonneuronal tissues, regulates the expression of ITPKA 2. But the mechanism for the increased ITPKA expression is complex and could be regulated by several molecular mechanisms including DNA methylation, microRNA, or aberrant transcription factor signaling. In the previous study from our lab, Liu et al. determined that the transcription factor TFAP2A promotes tumor metastasis and EMT via activating cytokeratin KRT16 transcriptionally 8, in the current study we identified TFAP2A could also activate the expression of ITPKA, which is a new mechanism for the ITPKA hyper-expression in lung adenocarcinoma.

In the current study, we reported ITPKA is upregulated in LUAD and associated with more aggressive clinical stages. ITPKA contributes to tumor proliferation, migration and cell death *in-vitro*, moreover, we provide evidence that TFAP2A transcriptionally induce the hyper-expression of ITPKA. Mechanistically, ITPKA executed its action through induction of EMT pathway and interaction with Drebrin 1. Last, in our survival analysis, the results indicate that the increased expression of ITPKA is associated with poor prognosis in LUAD patients.

## Results

### ITPKA is over-expressed in lung adenocarcinoma (LUAD) and correlates with lymph nodes metastasis

**Figure 1:**
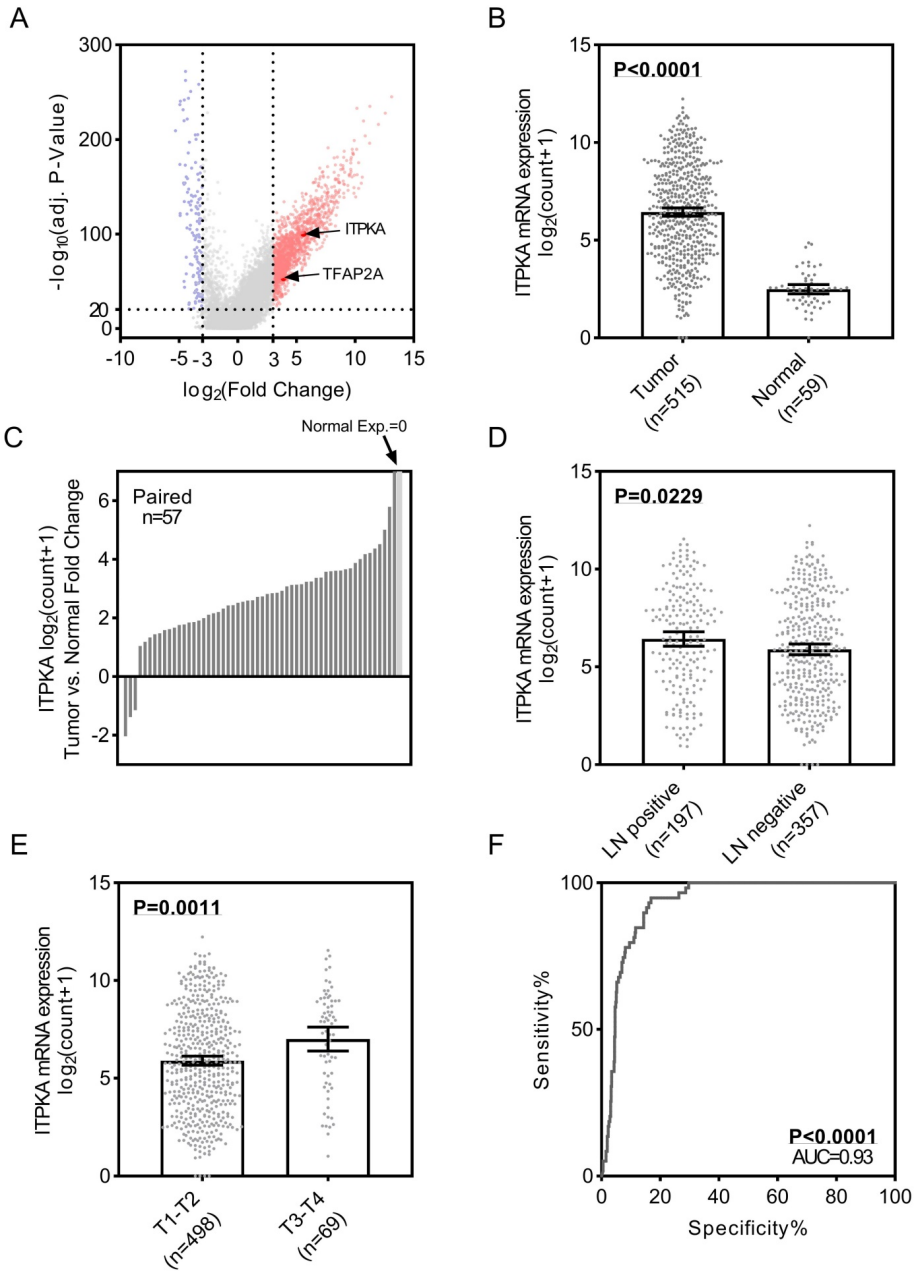
ITPKA is over-expressed in lung adenocarcinoma (LUAD) and correlates with lymph nodes metastasis. **A,** volcano plot for differential expressed genes in lung adenocarcinoma (LUAD) TCGA dataset, left blue part were genes significantly downregulated in LUAD and right red part were genes significantly upregulated in LUAD, and ITPKA and TFAP2A was at a relatively hyper-expression position. **B & C,** ITPKA was hyper expressed in LUAD compared with normal adjacent tissues, p<0.0001, and in the 57 paired samples, there were 2 pairs with null ITPKA expression in the normal tissues (but not their counterpart adjacent tumor samples). **D,** lymph node positive patients had higher ITPKA expression, p=0.0229. **E,** the ITPKA expression were higher in advanced T stage patients, p=0.0011. **F,** receiver operating characteristic curve (ROC) shown ITPKA expression had a good specificity and sensitivity for diagnosis in LUAD, p<0.0001, area under curve (AUC)=0.93.

To identify the differential expression of ITPKA in LUAD, we first analyzed the TCGA_LUAD dataset which containing 511 tumor samples and 58 normal samples (57 pairs) with clinical parameters. The bird view from the volcano plot revealed that both ITPKA and TFAP2A are at a relatively significantly differential expression position from 20475 genes (Fig 1A & B). The red dots represent the significantly hyper-expressed genes in LUAD. On the opposite, these blue dots represent the significantly hypo-expressed genes in LUAD (Fig 1A). Among the 57 paired tissues (LUAD tumor tissues vs. their adjacent normal tissues), there are 2 normal tissues with null ITPKA expression but not their adjacent LUAD tissues (Fig 1C). Moreover, the ITPKA expression is significantly higher in lymph node positive and more aggressive T stage LUAD tissues, indicating that ITPKA might contribute to the tumor progression and metastasis (Fig 1D & E). Last, the receiver operating characteristic curve (ROC) indicates that ITPKA expression could serve as a diagnostic marker for LUAD (Fig 1F).

### The biological function of ITPKA in lung adenocarcinoma (LUAD) cell proliferation, migration and cell death

**Figure 2:**
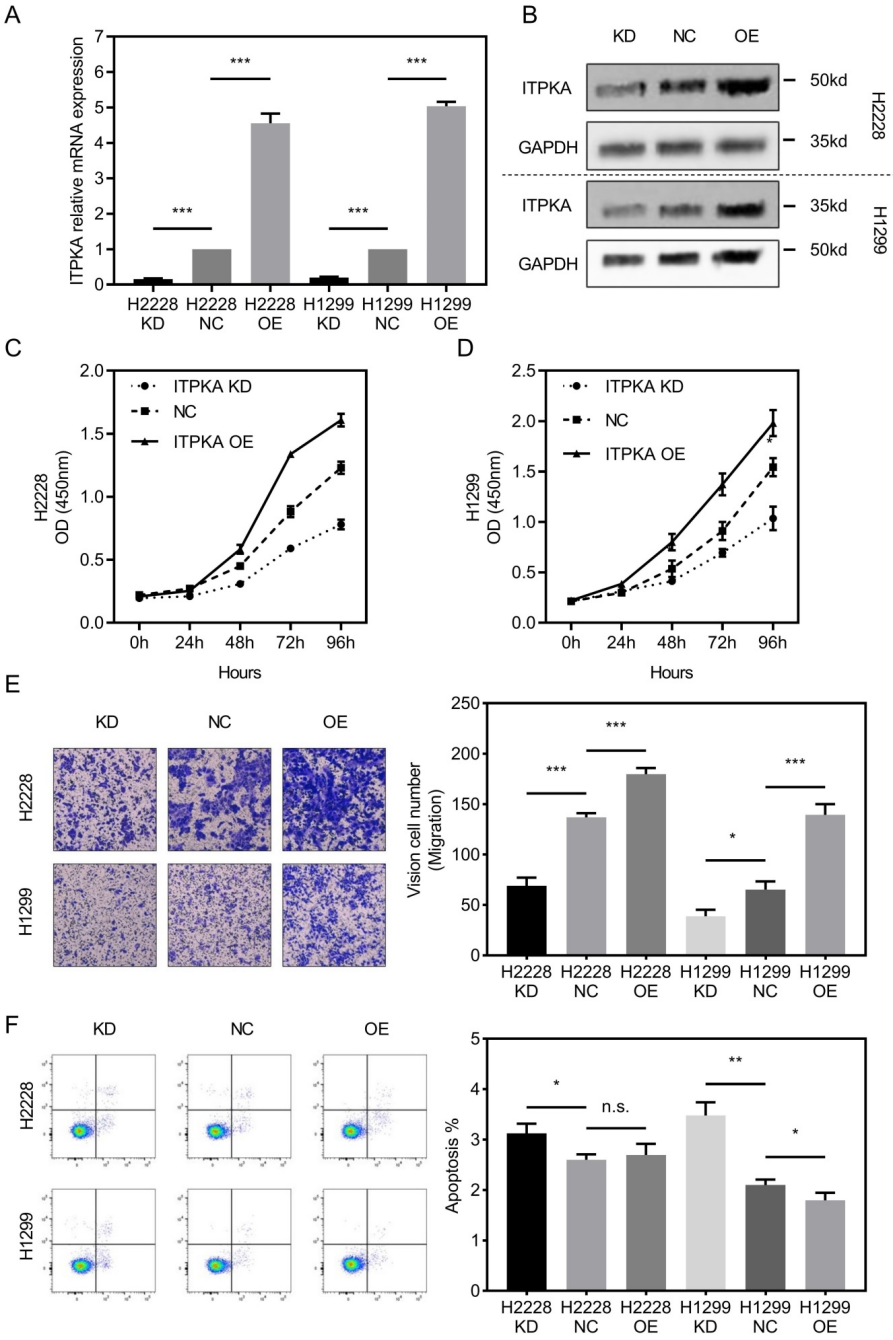
ITPKA influences proliferation, migration and cell death in LUAD cells. **A & B,** ITPKA was knocked down or over expressed, both shRNA and overexpression plasmid could significantly knockdown and overexpress the ITPKA mRNA expression and protein level in H2228 and H1299 cell lines. **C & D,** ITPKA knockdown group had lower proliferation rate and ITPKA overexpression group had higher proliferation rate. **E,** trans-well assay shown ITPKA could influence the migration ability in H2228 and H1299. **F,** ITPKA knockdown decrease the apoptosis rate in H1299 and H2228 cells and ITPKA overexpression could increase the apoptosis rate in H1299 cells.

To investigate the biological function of ITPKA *in-vitro*, ITPKA was knockdown or overexpressed in H1299 and H2228 cell lines. Both shRNA and overexpression constructs were able to effectively knockdown and overexpress ITPKA mRNA expression and protein level (Fig 2A & B). As shown in Fig 2C & D, the cell count kit 8 (CCK8) assay showed that the cell counts of these two cell lines were decreased by ITPKA knockdown and increased by ITPKA overexpression. In Fig 2E, the transwell assay revealed that ITPKA knockdown reduced migration ability and ITPKA overexpression increased the migration ability in both H1299 and H2228 cells. Last we determined the apoptosis rate in H1299 and H2228 cell lines, we found ITPKA knockdown could increase the apoptosis rate in both cell lines and ITPKA overexpression could decrease the apoptosis rate in H1299 cells (Fig 2F). In summary, the ITPKA has impacts on lung adenocarcinoma cell proliferation, migration, and cell death.

### ITPKA is involved with epithelial mesenchymal transition (EMT) in LUAD

**Figure 3:**
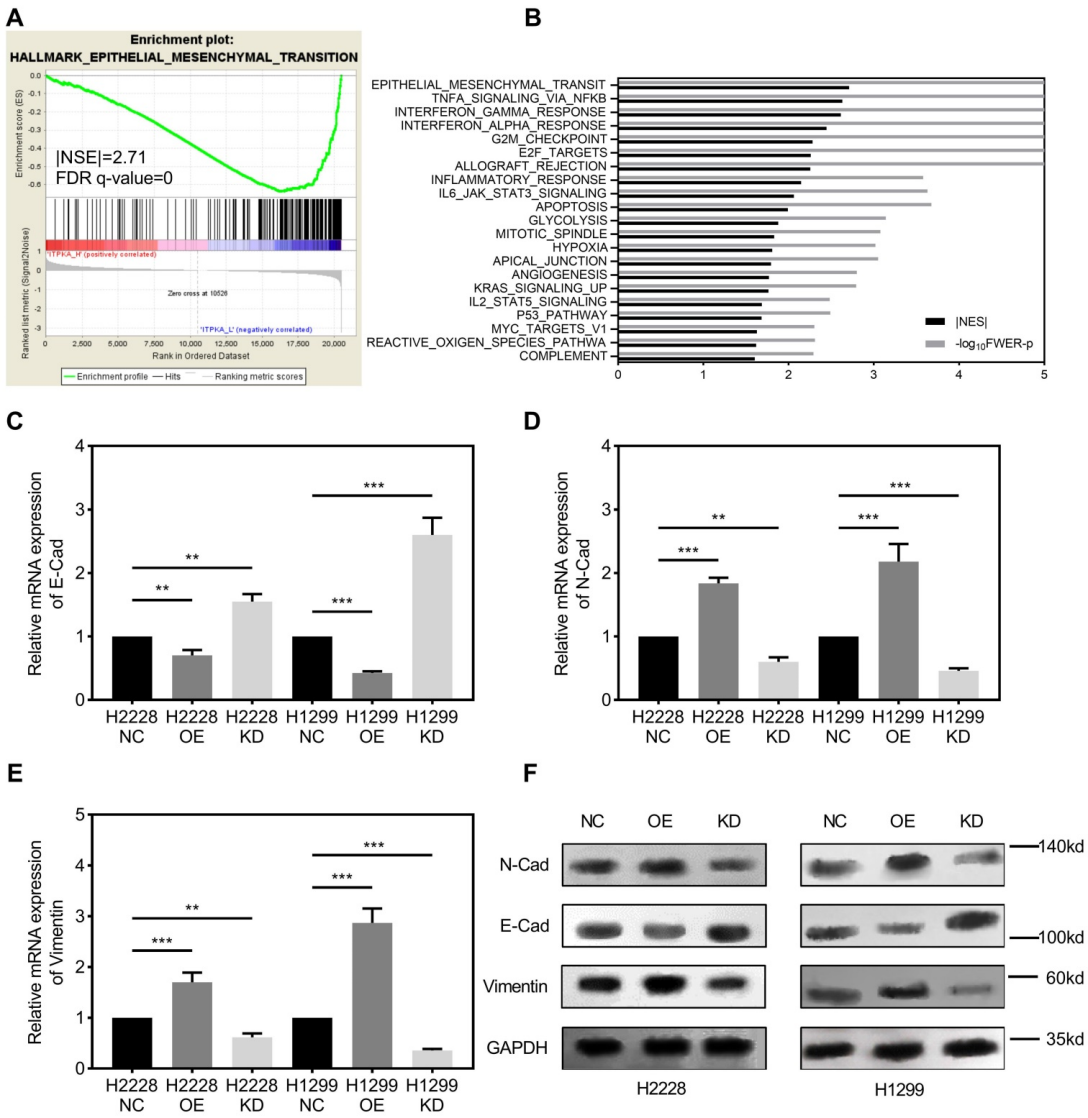
ITPKA is involved in epithelial mesenchymal transition (EMT). **A & B,** the TCGA_LUAD dataset (Top 10% ITPKA expression vs. Button 10% ITPKA expression) had been submitted for the Gene Set Enrichment Analysis (GSEA), these results indicating ITPKA was correlated with epithelial mesenchymal transition (EMT), Normalized Enrichment Score (NES)= 2.71, FDR<0.001. **C, D, E & F,** EMT markers were measured by qPCR and western blot, ITPKA knockdown group had lower N-Cad and Vimentin but higher E-Cad compared with their negative control group. And ITPKA overexpression groups indicated adverse results.

Gene set enrichment analysis. GSEA was introduced for potential mechanism exploring 8-10, and the transcriptome data from 50 TCGA_LUAD tumor tissues were submitted for GSEA (based on ITPKA expression, top 25 (top 10%) versus bottom 25 (bottom 10%) from 511 TCGA_LUAD tumor samples). These results indicate that epithelial mesenchymal transition (EMT) might be the downstream mechanism for ITPKA's phenotype in LUAD (Fig 3A & B). Given our findings that ITPKA expression is higher in LN positive tumors and ITPKA has impacts on LUAD cell migration, we sought to determine the expression of several EMT markers. Consistent with enrichment analyses, compared with NC, ITPKA knockdown have reduced N-Cad and Vimentin, and increased E-Cad, moreover, ITPKA overexpression increased the level of N-Cad and Vimentin, and decreased the level of E-Cad (Fig 3C-F). These data indicated that ITPKA knockdown influences EMT in LUAD.

### TFAP2A is the upstream transcription factor for ITPKA and ITPKA contributes to the oncogenic function of TFAP2A

**Figure 4:**
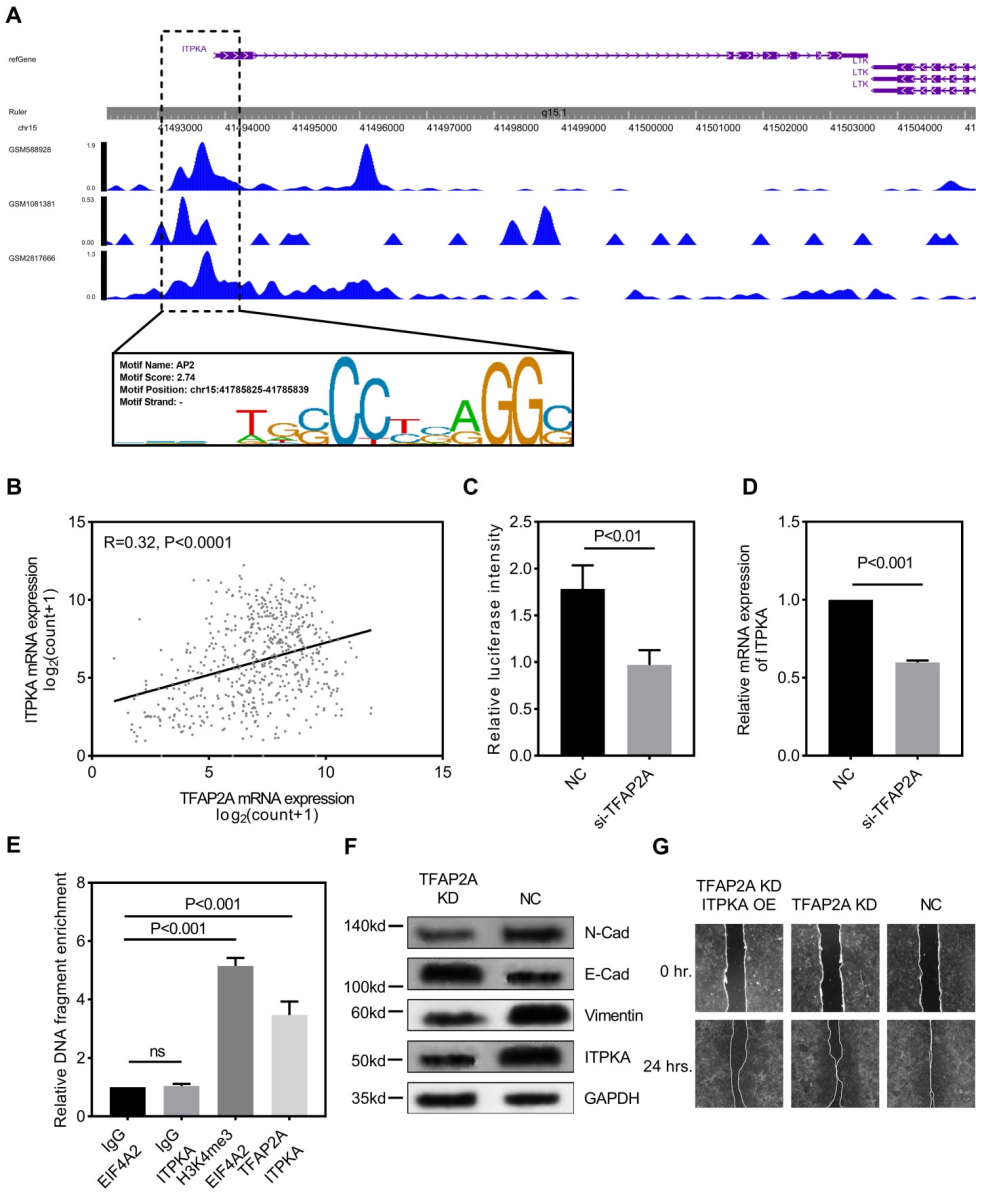
TFAP2A is the upstream transcription factor for ITPKA. **A,** CHIP-Seq peaks from three independent experiments indicate TFAP2A could bind on the core promotor region of ITPKA with an AP2 motif on chr15:41785825-41785839. **B,** TCGA_LUAD data indicates the expression of TFAP2A and ITPKA was positively corelated (Pearson R=0.32, P<0.0001). **C,** a luciferase reporter plasmid containing the core promotor sequence of ITPKA was utilized, after TFAP2A knockdown the relative luciferase intensity was decreased (a plasmid constantly expressing Renilla luciferase was used as a reference background signal) in H1299 cells. **D,** TFAP2A knockdown decreased the ITPKA expression in H1299. **E,** ChIP-qPCR indicated TFAP2A pull-down could enrich ITPKA promoter sequence in H1299 cells. **F,** TFAP2A knockdown H1299 cells have lower ITPKA, Vimentin and N-Cad level and higher E-Cad level. **G,** TFAP2A siRNA and/or ITPKA overexpression vector was co-transfected into H1299 cells, the ITPKA overexpression could partially rescue the migration phenotype in TFAP2A knockdown H1299 cells.

We explored the potential mechanism for ITPKA hyper-expression in LUAD. One AP2 motif was identified on the core promotor region of the ITPKA, so we sought to analyze the chromatin immunoprecipitation sequencing (ChIP-Seq) data from transcription factor (TF) that could bind to the AP2 motif. As shown in Fig 4A, three independent TFAP2A ChIP-Seq data (GSM2817666, GSM1081381, and GSM588928) all revealed a clear peak right on the core promotor of ITPKA. Moreover, the expression of TFAP2A is positively correlated with ITPKA (Fig 4B). By utilizing the duo-luciferase reporter system containing the core promotor region of ITPKA, we found that TFAP2A knockdown could significantly decrease the relative luciferase intensity (Fig 4C) and TFAP2A knockdown could also decrease ITPKA mRNA expression (Fig 4D) in H1299 cell line. After that, we performed ChIP-qPCR in H1299 cells for validating. Our results revealed that compared to negative control ITPKA promoter DNA fragments were enriched by TFAP2A antibody pull-down (Fig 4E).

Because we already reported TFAP2A is also significantly hyper-expressed in LUAD 8. We came to the conclusion that TFAP2A should be an important TF for ITPKA and contribute to the ITPKA hyper expression in LUAD. Because TFAP2A was reported involved in EMT 12, we measured EMT markers in H1299 cells with TFAP2A knockdown, as shown in Fig 4F, TFAP2A knockdown significantly reduced ITPKA, N-Cad, and Vimentin and increased E-Cad in H1299 cell line, so TFAP2A might exert its oncogenic actively through ITPKA via EMT. Last, we performed a rescue assay in which a plasmid containing the whole length of ITPKA was introduced, ITPKA overexpression could partially rescue the phenotype in TFAP2A knockdown H1299 cells (Fig 4G).

### ITPKA interacts with Drebrin 1, which contributes to its oncogenic function

**Figure 5:**
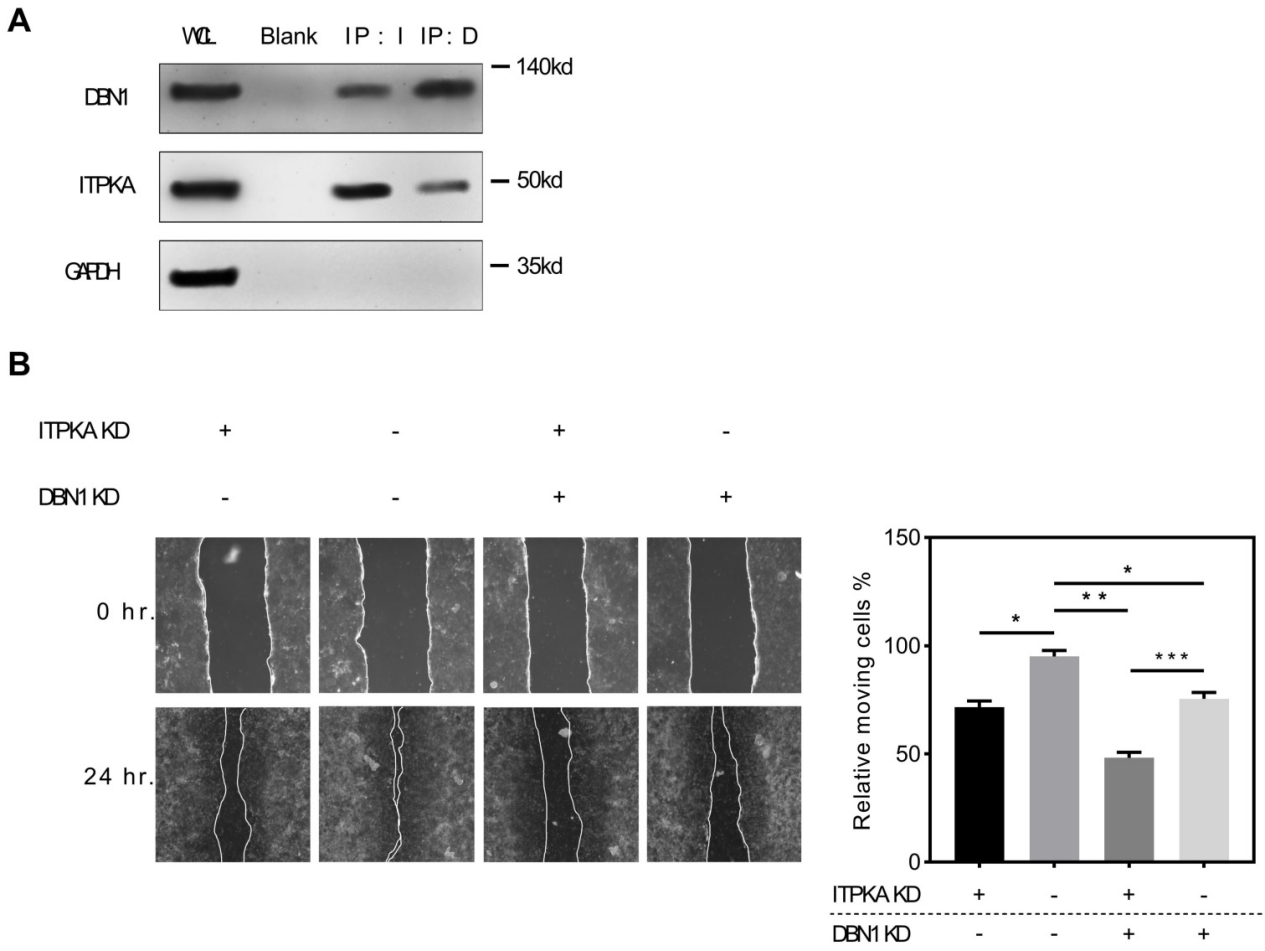
ITPKA interacts with Drbrin 1. **A,** the co-immunoprecipitation assay shown that DBN1 was able to be detected in anti-ITPKA antibody pull-down by from a total protein of H1299 cell; and in reverse co-immunoprecipitation assay, the purified pull-down protein by anti-DBN1 protein were detected by ITPKA antibody. The detection of GAPDH was used as a negative control. **B,** in wound healing assay, DBN1 and ITPKA knockdown could both undermine the migration ability in H1299 cells, and the double knockdown displayed the least migration ability in H1299 cells.

To explore the potential interaction with ITPKA we screened public available mass spectrometric data 11, we found ITPKA might interact with DBN1 (Drebrin 1), to validate this potential interaction, we performed the Co-IP (co-immunoprecipitation) assay, briefly the co-immunoprecipitation complex that was pulled down by anti-ITPKA antibody from a total protein of H1299 cell line were detected by a DBN1 antibody, we found DBN1 was detected; for further validation we performed a reverse co-immunoprecipitation assay, the purified pull-down protein by anti-DBN1 protein were detected by ITPKA antibody. The detection of GAPDH was used as a negative control. As shown in Fig 5A, in DBN1 pull down ITPKA was detectable and in ITPKA pull-down, DBN1 was also detectable; from these data, we propose that ITPKA interacts with DBN1. For exploring the biological function of DBN1 with ITPKA, we utilized shRNAs which could knockdown the DBN1 and ITPKA, as shown in Fig 5B, DBN1 and ITPKA knockdown could both undermine the migration ability of H1299 cells, and the double knockdown displayed the least migration on H1299 cells, indicating DBN 1 contributed to the biological function of ITPKA. In summary, our data indicate DBN1 interacts with ITPKA and contributes to its biological function.

### ITPKA hyper expression predicts poor prognosis in LUAD

**Figure 6:**
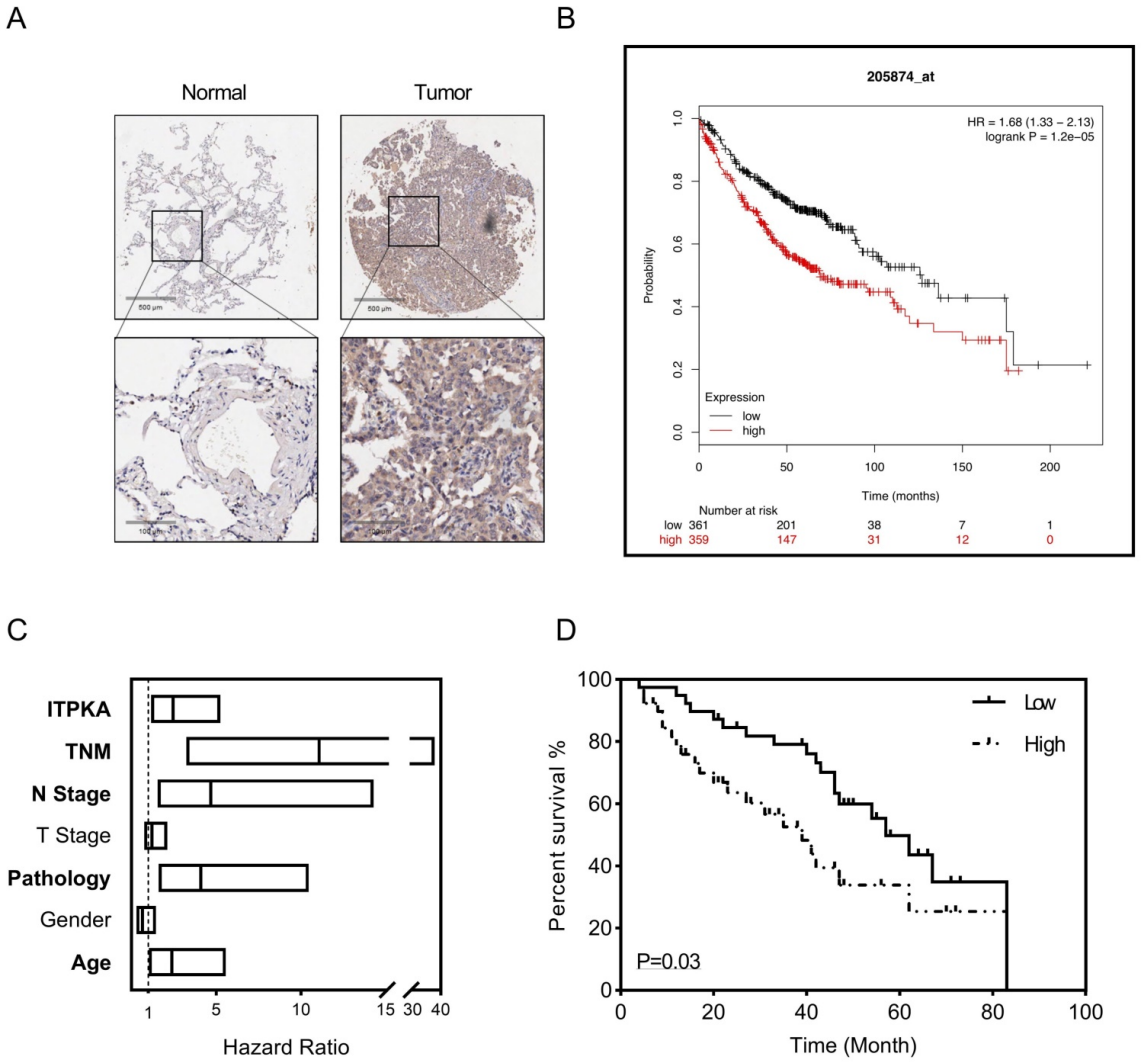
ITPKA hyper expression predicts poor prognosis in LUAD. 92 patients were enrolled in the multivariant cox regression survival analysis. **A,** compared with tumor samples the normal samples have lower ITPKA level in IHC. **B,** the K-M plot form http://kmplot.com indicates that ITPKA is negatively corelated with LUAD patients survival, HR=1.68(1.33-2.13). **C,** the forest plot of multivariant cox regression analysis shown TNM stage, N stage, pathology, age and ITPKA level could serve as independent indicators for survival (data in Table S1). **D,** ITPKA staining score was positively corelated with prognosis (HR= 2.467(1.16-5.25), p=0.03, multivariant cox regression).

To explore the clinical significance of ITPKA, a tissue microarray described before were introduced 8, the LUAD tissue microarray (TMA) contain 92 tumor samples with follow up survival data, after staining with ITPKA, in the multivariant cox regression survival analysis was performed, several clinical parameters including age, gender, pathology, T stage, N stage, TNM stage and ITPKA level were enrolled. As shown in Fig 6A, in normal lung tissues the ITPKA expression is very low, but LUAD tissues have higher ITPKA level, and the open access survival data from km-plot (http://kmplot.com) indicates higher ITPKA mRNA expression predicts worse prognosis (Fig 6B) 12. In cox regression survival analysis from the TMA, we found age, pathology, N stage, TNM stage and ITPKA level could serve as independent survival markers in LUAD patients (Fig 6C & D), And last, in Table 1, high level of ITPKA was corrected with lymph node positive in LUAD patients.

## Methods

### Data sources

The level 3 TCGA lung adenocarcinoma datasets containing 511 tumors and 58 adjacent normal lung tissues (57 paired tissues) were downloaded for clinical analysis, the “count” is the RSME normalized mRNA count 13, which represents the mRNA expression of genes. For volcano plot, F-test was used between tumor and normal in 20475 genes. And in receiver operating characteristic curve (ROC), the ITPKA gene expression unit is “log_2_(count+1)”. For GSEA, the latest official tool was downloaded from http://software.broadinstitute.org/gsea (Ver. 3.0).

### Cell lines, cell culture, and plasmids

H2228 cells and H1299 cells were obtained from American Type Culture Collection (ATCC, USA). Cells were cultured in RPMI1640 media (Kaiji, Nanjing, China) supplemented with 10% fetal bovine serum and 1% penicillin/streptomycin and cultured at 37°C in a humidified incubator containing 5% CO2. Transfection was followed by instruction of Lipofectamine 3000 (Invitrogen, USA). The human ITPKA targeting small hairpin RNA (shRNA) was designed with the targeting sequence 5′-CCUUGUGUGCUCGACUGCA-3′ adopted from a published paper^14^, and the DBN1 shRNA with 5′-GGAUUAACCGAGAGCAGUU-3′ targeting sequence was also introduced^15^, the two shRNA were inserted into pGFP-C-shLenti vector for lentivirus packaging, and the expression plasmid with full length of ITPKA and the luciferase reporter plasmids (firefly luciferase) containing the core promotor region of ITPKA were both customized and ordered from RiboBio (Guangzhou, China); and a Renilla luciferase expression plasmid was used for background control, the method for determine the luciferase activity were described before^8^.

### Cell proliferation, migration, and wound healing assay

The Cell Counting Kit-8 (KGA317; KeyGen Biotech, Nanjing, China) was used to determine the cell proliferation rate. 2,000 cells/well (100 µl total volume) were seeded in 96-well plates and the absorbance was measured at 450 nm using a plate Reader (Promega, GM3000). The experiment was repeated in triplicate at various time points for 4 days. For migration assays, transfected cells (40,000 cells/well; 100 µl total volume) were seeded in the upper chamber of Transwell assay inserts (8-mm pores; Millipore) containing 200 µl serum-free media. The lower chambers were filled with same media containing 10% FBS. After 24 hours in incubation, cells were fixed with methanol and stained with crystal violet. Cell migration or invasion was assessed by counting the number of stained cells in five random fields of each group. In wound healing assay, cells were seeded and transfected in six-well plates, after 24 hours an artificial scratch wound on a confluent monolayer of cells was created with a 200μl pipette tip, then serum-free medium was added, cells were imaged at baseline and 24 hours. Experiments were repeated in triplicate.

### Flow-cytometry analysis

Flow-cytometry analysis was performed for detecting apoptosis rate. Briefly, cells were washed and resuspended at a concentration of 1 × 10^6^ cells/ml, then cells were stained with 7-AAD (Invitrogen, A1310) and Annexin V (eBioscience, BMS306FI-100). After 20min incubation at room temperature, cells were analyzed by a BD FACSCanto™ II flow cytometer. Each assay was performed in triplicate.

### RNA preparation, reverse transcription, real-time quantitative PCR, and ChIP-qPCR

TRIzol was used for extracting total RNA from cultured cells, for qPCR 1µg total RNA was reverse transcribed to 20µl cDNA by ipsogen RT Kit (QIAGEN, Cat No: 679913). qPCR was performed with SYBR Select Master Mix (Applied Biosystems, Cat: 4472908). For ChIP-qPCR DNA preparation, the EpiTect ChIP OneDay Kit (QIAGEN, Cat No: 334471) was used based on official user guide, rabbit IgG (#PP64, Millipore) as the negative control, and H3K4me3 pull down as a positive control (the sequence of EIF4A2 was detected) and IgG pull down as negative control. The qPCR was performed using QuantStudio^TM^ 3 Real-Time PCR System; the reaction protocol was 95 °C for 10 min, followed by 40 cycles of 92°C for 15 sec and 62.5°C for 1 min. Every sample was in triplicate and the expression was normalized to the reference gene expression (hGAPDH) using the 2^-ΔΔCt^ method, and for ChIP enrichment fold change, samples were normalized to the IgG pulldown group and 2^-(CtTarget - CtIgG)^. All the primers for qPCR are shown in supplementary file Table S1.

### Protein preparation, western blot, Immunohistochemistry and co-immunoprecipitation

The total protein was extracted using RIPA lysis buffer (Beyotime Biotechnology, P0013C) supplemented with protease inhibitors cocktail (Promega, G6521), protein concentration was measured by BCA kit (Beyotime Biotechnology, P0010S). After that as a standard western blot protocol, proteins were loaded and separated by 12% SDS-PAGE gel electrophoresis and incubated with primary and HRP-tagged secondary antibodies to detect the signal (antibody list in supplementary file Table S2). For co-immunoprecipitation (co-IP), a commercially available co-immunoprecipitation Kit (Pierce, Lot. 26149) was used to investigate a potential interaction between ITPKA and DBN1 following the official instruction. The normal serum IgG was used as negative control. Western blotting was used to determine the presence of ITPKA, DBN1 or GAPDH in the co-immunoprecipitated complex.

The tissue microarray (TMA) containing 88 cases of lung adenocarcinoma (LUAD) with matched adjacent normal tissue and 4 cases only have tumors were obtained from Shanghai Biochip Co., Ltd. (Cat. No. HLugA180Su03)^8^. This research was approved by the Human Research Ethics Committee of Nanjing Medical University. For IHC staining, briefly the slides were incubated with anti-human ITPKA antibody overnight at 4 °C, then sides were incubated using the secondary antibody for 2 hours in room temperature. After that the immunohistochemistry results were scored based on the method described before^8^.

### Statistical analysis

Data are presented as the mean ± standard deviation and analyzed using a Student's t-test or one-way ANOVA with post-hoc analysis. P<0.05 was considered to indicate a statistically significant difference. SPSS Statistics (version 20.0, IBM Corp), Excel (Office 365, Microsoft Office) and GraphPad Prism (version 7.05, GraphPad Software, Inc) software were used for statistical analyses and the production of graphs.

## Discussion

In the current study, we present evidence that ITPKA is over-expressed in LUAD and the over-expression of ITPKA is associated with lymph node metastasis and more advanced T stage. ITPKA expression also has good specificity and sensitivity for LUAD diagnosis. By using LUAD cell lines, we determined ITPKA knockdown inhibits cell proliferation, migration, and apoptosis, which contributes to less aggressive phenotypes, and ITPKA overexpression contributes to more aggressive phenotypes. These indicate ITPKA plays an important role in tumorigenesis and progress in LUAD. For downstream mechanism exploring, the GSEA analysis was introduced, by submitting TCGA data for GSEA we found ITPKA was involved in EMT. Thus, several EMT markers were measured between ITPKA knockdown, overexpression, and negative control, we found that ITPKA knockdown leads to higher E-Cad level but lower N-Cad and Vimentin level and ITPKA overexpression shown opposite results.

Previously we reported the transcription factor TFAP2A could induce the hyper-expression of KRT16 8. In the current study, we further reported that TFAP2A could also induce the expression of ITPKA. The promotor region of ITPKA contains an AP2 motif which has the potential to bind with transcription factor TPAP2A, TFAP2A is hyper-expressed in LUAD and influencing EMT 8, 16 and also positively correlated with ITPKA expression. By using three independent published ChIP-Seq data (GSM2817666, GSM1081381, and GSM588928), we found that in all three datasets TFAP2A could bind to the core promotor region of ITPKA, this indicates TFAP2A could be a transcription factor for ITPKA. To validate whether TFAP2A could transcriptionally activate ITPKA expression, we designed the duo-luciferase system containing the sequence of ITPKA core promotor sequence. We found that TFAP2A knockdown could significantly decrease the relative luciferase intensity; moreover, for better validation, ChIP-qPCR was performed, indicating that the ITPKA promoter sequence was enriched in the TFAP2A pull-down DNA fragments. As a result, the transcription factor TFAP2A could induce the hyper-expression of ITPKA in LUAD. As it's reported, TFAP2A influences tumor migration via EMT 8, 16, 17. We designed the migration rescue assay. We found that TFAP2A knockdown could influence EMT and decrease the cancer cells' migration in LUAD. But after we overexpressed ITPKA in the TFAP2A knockdown cells, we found the migration phenotype was partly rescued in LUAD cells, indicating TFAP2A could exert its oncogenic ability via ITPKA.

Weak interactions shape the cellular protein-protein interaction network, which is important for the cellular biological function, to explore the potential interaction with ITPKA we screened public available mass spectrometric data 11, we found ITPKA might interact with DBN1 (Drebrin 1), which is an F-actin related protein that remodels actin to facilitate the change of filopodia into dendritic spines during synaptogenesis in developing neurons 18, moreover DBN1 expression exhibits potential clinical significance in cancer patients and related with metastasis 19, 20. To validate this potential interaction, we performed the co-immunoprecipitation assay, we found that in anti-ITPKA antibody pull-down DBN1 can be detected, and in reverse co-immunoprecipitation assay ITPKA was also detected in anti-DBN1 pull down. For functional validation, we knockdown the DBN1 and/or ITPKA in LUAD cells, we found DBN 1 interacts with ITPKA and they both contributed to the wound healing ability in LUAD cells. Last, survival analysis on human LUAD sample TMA (with follow up data) was performed. The multivariate cox regression indicates LUAD patients with strongly expressed ITPKA have a poorer prognosis than those with absent or weaker ITPKA expression.

Our data indicate ITPKA hyperexpression in LUAD is induced by transcription factor TFAP2A. ITPKA could influence cancer cell migration, invasion, and proliferation via EMT, and interacting with DBN1. And ITPKA may serve as an independent prognosis maker in LUAD.

## Supplementary Material

Supplementary figures and tables.

## Figures and Tables

**Table 1:** 

	Groups	ITPKA		Pearson χ2	P-value
		High	Low		
Gender	Male	27	24	0.40	0.53
	Female	19	22		
Age	≤60	17	21	0.72	0.40
	>60	29	25		
Differentiation	Well	29	32	0.44	0.51
	Moderate-Poor	17	14		
T stage	T1-T2	27	33	3.17	0.08
	T3-T4	13	6		
Lymph node^*^	Negative	13	21	5.67	**0.02**
	Positive	24	12		
TNM stage	I-II	19	22	2.32	0.13
	III-IV	17	9		

P<0.05 was considered as significant, *Significant correlation.

## Abbreviations

LUAD: Lung adenocarcinoma
EMT: Epithelial-mesenchymal transition
GSEA: Gene set enrichment analysis
IHC: Immunohistochemistry
qRT-PCR: Quantitative real-time polymerase chain reaction
TCGA: The Cancer Genome Atlas
ChIP: Chromatin immunoprecipitation
ROC: Receiver operating characteristic curve
NES: Normalized enrichment score

## References

[1] Schell MJ (2010). Inositol trisphosphate 3-kinases: focus on immune and neuronal signaling. Cell Mol Life Sci.

[2] Chang L, Schwarzenbach H, Meyer-Staeckling S, Brandt B, Mayr GW, Weitzel JM (2011). Expression Regulation of the Metastasis-Promoting Protein InsP3-Kinase-A in Tumor Cells. Mol Cancer Res.

[3] Johnson HW, Schell MJ (2009). Neuronal IP3 3-kinase is an F-actin-bundling protein: role in dendritic targeting and regulation of spine morphology. Mol Biol Cell.

[4] Windhorst S, Song K, Gazdar AF (2017). Inositol-1,4,5-trisphosphate 3-kinase-A (ITPKA) is frequently over-expressed and functions as an oncogene in several tumor types. Biochem Pharmacol.

[5] Li J, Zhu YH, Huang P, Zhang B, Sun J, Guan XY (2015). ITPKA expression is a novel prognostic factor in hepatocellular carcinoma. Diagn Pathol.

[6] Wang YW, Ma X, Zhang YA, Wang MJ, Yatabe Y, Lam S (2016). ITPKA Gene Body Methylation Regulates Gene Expression and Serves as an Early Diagnostic Marker in Lung and Other Cancers. J Thorac Oncol.

[7] Windhorst S, Kalinina T, Schmid K, Blechner C, Kriebitzsch N, Hinsch R (2011). Functional role of inositol-1,4,5-trisphosphate-3-kinase-A for motility of malignant transformed cells. Int J Cancer.

[8] Yuanhua L, Pudong Q, Wei Z, Yuan W, Delin L, Yan Z (2019). TFAP2A Induced KRT16 as an Oncogene in Lung Adenocarcinoma via EMT. Int J Biol Sci.

[9] Subramanian A, Tamayo P, Mootha VK, Mukherjee S, Ebert BL, Gillette MA (2005). Gene set enrichment analysis: a knowledge-based approach for interpreting genome-wide expression profiles. Proc Natl Acad Sci U S A.

[10] Mootha VK, Lindgren CM, Eriksson KF, Subramanian A, Sihag S, Lehar J (2003). PGC-1alpha-responsive genes involved in oxidative phosphorylation are coordinately downregulated in human diabetes. Nat Genet.

[11] Hein MY, Hubner NC, Poser I, Cox J, Nagaraj N, Toyoda Y (2015). A human interactome in three quantitative dimensions organized by stoichiometries and abundances. Cell.

[12] Nagy A, Lanczky A, Menyhart O, Gyorffy B (2018). Validation of miRNA prognostic power in hepatocellular carcinoma using expression data of independent datasets. Sci Rep.

[13] Li B, Dewey CN (2011). RSEM: accurate transcript quantification from RNA-Seq data with or without a reference genome. BMC Bioinformatics.

[14] Gilot D, Le Meur N, Giudicelli F, Le Vee M, Lagadic-Gossmann D, Theret N (2011). RNAi-based screening identifies kinases interfering with dioxin-mediated up-regulation of CYP1A1 activity. PLoS One.

[15] Dart AE, Worth DC, Muir G, Chandra A, Morris JD, McKee C (2017). The drebrin/EB3 pathway drives invasive activity in prostate cancer. Oncogene.

[16] Zhang D, Li H, Jiang X, Cao L, Wen Z, Yang X (2017). Role of AP-2alpha and MAPK7 in the regulation of autocrine TGF-beta/miR-200b signals to maintain epithelial-mesenchymal transition in cholangiocarcinoma. J Hematol Oncol.

[17] Huang W, Chen C, Liang Z, Qiu J, Li X, Hu X (2016). AP-2alpha inhibits hepatocellular carcinoma cell growth and migration. Int J Oncol.

[18] Mancini A, Sirabella D, Zhang W, Yamazaki H, Shirao T, Krauss RS (2011). Regulation of myotube formation by the actin-binding factor drebrin. Skelet Muscle.

[19] Mitra R, Lee J, Jo J, Milani M, McClintick JN, Edenberg HJ (2011). Prediction of postoperative recurrence-free survival in non-small cell lung cancer by using an internationally validated gene expression model. Clin Cancer Res.

[20] Lin Q, Tan HT, Lim TK, Khoo A, Lim KH, Chung MC (2014). iTRAQ analysis of colorectal cancer cell lines suggests Drebrin (DBN1) is overexpressed during liver metastasis. Proteomics.

