# Supplementary material for: TFAP2A Induced ITPKA Serves as an Oncogene and Interacts with DBN1 in Lung Adenocarcinoma
Source: Int J Biol Sci. 2020 Jan 1;16(3):504–14. doi: 10.7150/ijbs.40435 (PMC6990902; doi:10.7150/ijbs.40435)
Supplement: Supplementary file 1 — Supplementary figures and tables. [file ijbsv16p0504s1.pdf]

sTable 1, primer list, providing the qPCR primers in this paper.

qPCR	Forward Primer	Reverse Primer
ITPKA	CCTTTCCACCTCGTCGGTCT	GCCTTAAAACTCCCAGTGTGC
GAPDH	GGAGCGAGATCCCTCCAAAAT	GGCTGTTGTCATACTTCTCATGG
E-Cad	ATTTTTCCCTCGACACCCGAT	TCCCAGGCGTAGACCAAGA
N-Cad	TCAGGCGTCTGTAGAGGCTT	ATGCACATCCTTCGATAAGACTG
Vimentin	AGTCCACTGAGTACCGGAGAC	CATTTCACGCATCTGGCGTTC
TFAP2A	GACTCGGAGACCTCTCGATCC	GACGGCATTGCTGTTGGAC
ChIP-qPCR	Forward Primer	Reverse Primer
ITPKA	TAGGGGACCAAGAGTAGGAC	CCCACTCCTTCCACTTTCAG
EIF4A2	TTTTGTAGCTGACCGAAGCA	GCGCCCTATGACCTTCACTA

sTable 2, the antibody list, providing all the antibodies used in this paper.

Target	Lot. No.	Supplier
DBN1	ab60933	Abcam
ITPKA	14270-1-AP	Proteintech
E-Cad	CST	#3195
N-Cad	CST	#13116
Vimentin	CST	#5741
GAPDH	Santa Cruz	sc-47724
H3K4me3	Abcam	ab8580
TFAP2A	Abcam	ab52222
Anti-mouse IgG	CST	#14709
Anti-rabbit IgG	CST	#14708

A

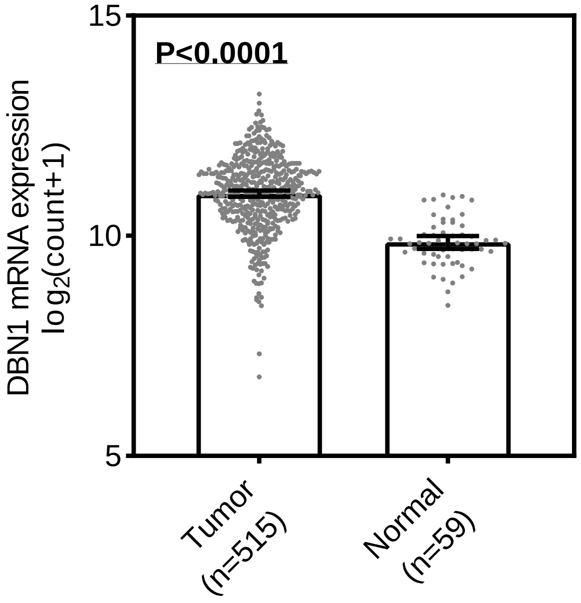
P<0.0001
DBN1 mRNA expression
$\log_2$(count+1)
15
10
5
Tumor
(n=515)
Normal
(n=59)

B

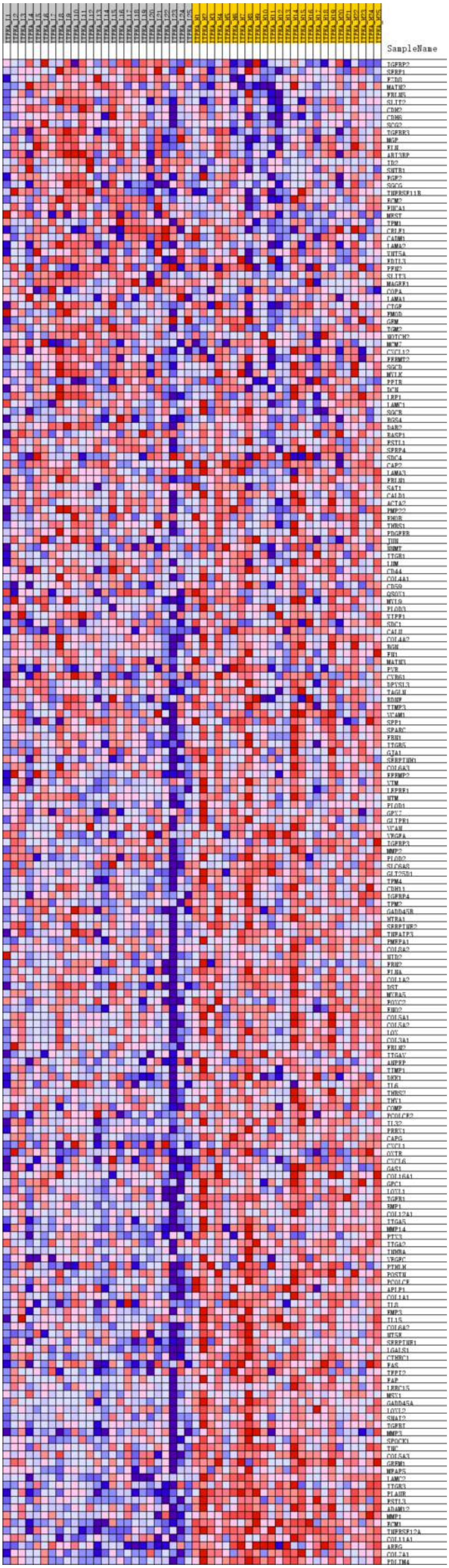
SampleName
